# Ponatinib plus asciminib in myeloid blast-phase CML harboring a phased *BCR*::*ABL1* Y253H/T315I compound mutation: a case report

**DOI:** 10.3389/fonc.2026.1928961

**Published:** 2026-08-20

**Authors:** Yingying Hu, Hongbing Ma, Xu He

**Affiliations:** 1 Department of Hematology, Hospital of Chengdu Office of the People's Government of Xizang Autonomous Region, Chengdu, Sichuan, China; 2 Department of Hematology, West China Hospital, Sichuan University, Chengdu, Sichuan, China

**Keywords:** asciminib, BCR::ABL1, case report, chronic myeloid leukemia, compound mutation, myeloid blast-phase CML, ponatinib

## Abstract

Compound *BCR*::*ABL1* mutations, particularly those including T315I in combination with P-loop substitutions, represent a major therapeutic challenge in advanced-phase chronic myeloid leukemia (CML). Clinical evidence supporting dual ATP-site and allosteric inhibition in this setting remains limited. We report a heavily pretreated patient with myeloid blast-phase CML in whom next-generation sequencing at blast transformation identified Y253H, T315I, and a low-level Y253_E255delinsHGK variant. Importantly, phasing analysis demonstrated that Y253H and T315I were present on the same *BCR*::*ABL1* allele, confirming a true compound mutation. Given the aggressive disease biology, limited therapeutic options, and transplant intent, the patient received ponatinib 45 mg once daily plus asciminib 200 mg twice daily as an individualized bridge strategy. After 4 weeks of treatment, bone marrow evaluation showed a transient reduction in blast burden. However, treatment exposure was brief and interrupted by pancreatitis, no molecular response was documented, and rapid relapse occurred before planned allogeneic transplantation. This case should therefore not be interpreted as evidence of efficacy. Rather, it provides a cautious, hypothesis-generating clinical observation suggesting possible short-term activity of dual ATP-site and allosteric inhibition in a highly resistant setting. This case also highlights the potential value of long-read sequencing-based mutation profiling with phasing in advanced-phase CML, where distinguishing true compound mutations from separate subclones may be clinically relevant.

## 1 Introduction

Chronic myeloid leukemia (CML) is a myeloproliferative neoplasm driven by the *BCR*::*ABL1* fusion kinase. The introduction of tyrosine kinase inhibitors (TKIs) has dramatically improved outcomes for patients with chronic-phase disease (1). However, the prognosis remains poor once CML progresses to the blast phase, particularly myeloid blast-phase CML, where rapid disease evolution, clonal complexity, treatment resistance, and limited durability of the response continue to pose major therapeutic challenges (2). In this setting, allogeneic transplantation remains the only potentially curative strategy for eligible patients, and achieving sufficient disease control to proceed to transplantation is often difficult (3).

Among the mechanisms of TKI resistance, *BCR*::*ABL1* kinase domain mutations are of particular clinical importance. While single mutations have long been recognized, the increasing use of next-generation sequencing (NGS) has revealed a more complex mutational landscape, including compound mutations, defined as two or more mutations occurring on the same *BCR::ABL1* allele (4). This distinction is clinically relevant because true compound mutations may confer resistance patterns that differ substantially from those of separate subclones. In particular, T315I-inclusive compound mutations, especially those combined with P-loop substitutions such as Y253H, have been associated with marked resistance to currently available TKIs in preclinical studies and represent among the most difficult therapeutic scenarios in CML (5).

Ponatinib retains activity against many single *BCR::ABL1* resistance mutations, including isolated T315I (6), whereas asciminib inhibits *BCR::ABL1* through a mechanistically distinct allosteric interaction with the myristoyl pocket (7, 8). On this basis, combined ATP-site and allosteric inhibition has emerged as a biologically plausible strategy to suppress resistant mutant clones that may be inadequately controlled by either agent alone. Preclinical studies have suggested additive or synergistic activity for this approach, but clinical evidence remains limited, particularly in advanced-phase disease and in patients with phased compound mutations (9).

Here, we report a case of heavily pretreated myeloid blast-phase CML in which NGS identified Y253H, T315I, and a low-level Y253_E255delinsHGK variant, with phasing analysis confirming that Y253H and T315I were present on the same allele. The patient received ponatinib plus asciminib as an individualized bridge strategy before planned allogeneic transplantation. Because treatment exposure was brief and interrupted, no molecular response was documented, and relapse occurred rapidly; thus, this case should not be interpreted as proof of efficacy. Rather, it is presented as a cautious, hypothesis-generating clinical observation that highlights both the potential relevance of mutation phasing and the therapeutic challenges posed by compound-mutant blast-phase CML.

## 2 Case presentation

A 44-year-old man was diagnosed with chronic-phase chronic myeloid leukemia (CML-CP) in 2013 after evaluation for leukocytosis. The diagnosis was confirmed by bone marrow cytogenetic and molecular testing, which demonstrated a p210 (e14a2) *BCR*::*ABL1* transcript with the p190 transcript negative, and imatinib 400 mg once daily was initiated. He maintained a major molecular response (MMR; *BCR*::*ABL1* international scale [IS] ≤ 0.1%) from 2013 to 2018. In February 2018, the *BCR*::*ABL1* transcript level increased to 1.98% (IS), indicating the loss of MMR. Owing to financial constraints, treatment escalation was delayed, and nilotinib was started in June 2020. Serial BCR::ABL1 transcript data during this interval are not available, reflecting interrupted molecular monitoring across institutions.

In December 2023, he developed a right shoulder subcutaneous mass, disseminated cutaneous nodules, and left-sided hearing loss. Bone marrow examination showed marked myeloid hyperplasia with 9% blasts and combined eosinophilia of 18%. Biopsies of the shoulder mass and skin lesions were consistent with myeloid sarcoma. Immunohistochemistry showed positivity for CD117, myeloperoxidase (MPO), and CD43, partial positivity for CD34, a Ki-67 index of approximately 95%, and CD30 expression in approximately 80% of cells; CD3, CD79a, and CD56 were negative. In the context of established p210 *BCR*::*ABL1*-positive CML with a documented decade-long chronic phase, these findings were consistent with extramedullary involvement by myeloid blast-phase CML rather than *de novo* BCR::*ABL1*-positive AML. ABL1 kinase domain testing detected p.F317L and p.H396R. Intensive chemotherapy was recommended but declined, and nilotinib was continued.

Between February and March 2025, the cutaneous lesions progressed, and the percentage of bone marrow blasts increased to 17%. Clinical targeted next-generation sequencing (NGS) of bone marrow identified p.T315I and p.Y253H mutations in ABL1. Olverembatinib (40 mg every other day) was initiated on 28 March 2025, but the treatment was complicated by grade 4 myelosuppression.

By July 2025, the disease had progressed, with 64% peripheral blood blasts and 60% bone marrow blasts. In August 2025, azacitidine plus venetoclax was administered in combination with olverembatinib. The course was complicated by probable invasive pulmonary fungal infection on chest computed tomography and *Klebsiella pneumoniae* bloodstream infection. Bone marrow blasts decreased to 17% on 8 September and 14% on 18 September, but *BCR*::*ABL1*/*ABL1* (IS) remained 207%. Cytogenetic analysis showed a complex karyotype: 50,XY, + 8,+8,t (9,22),+der (22)t (9,22),+der(22)t(9;22) (16). After a second dose-reduced cycle, marrow blasts increased to 25% on 17 October, and infectious complications precluded further treatment intensification. Repeat NGS showed persistent ABL1 p.Y253H (variant allele frequency [VAF], 17.77%) and p.T315I (VAF, 6.62%), together with ABL1 Y253_E255delinsHGK (VAF, 4.18%) and ASXL1 (VAF, 13.08%). This clinical assay was a hybridization capture–based targeted next-generation sequencing panel covering 248 genes recurrently mutated in myeloid malignancies (Kindstar Global, Chengdu, China), sequenced on a DNBSEQ-T1+ platform to a mean target-region depth of ≥1000×.

Because the clinical NGS assay quantified the mutation burden but did not determine the allelic phase, residual bone marrow material was subsequently analyzed using long-read Oxford Nanopore sequencing with an approximately 10-kb amplicon spanning ABL1 exons 4-6. A total of 27,990 informative reads covered both mutation sites. Of these, 1,520 reads (5.4%) harbored both Y253H and T315I on the same molecule, whereas no reads containing T315I in the absence of Y253H were detected (0/27,990). These findings confirmed that Y253H and T315I were present in cis on the same ABL1 allele, consistent with a true compound mutation.

Given refractory CML in the myeloid blast phase with extramedullary disease, complex cytogenetic abnormalities, and a confirmed cis ABL1 compound mutation, ponatinib 45 mg once daily plus asciminib 200 mg twice daily was initiated on 29 October 2025 (day 1), Given the known toxicity profiles of both agents, a structured monitoring protocol was implemented, comprising complete blood counts two to three times weekly; liver and pancreatic enzyme panels (including amylase and lipase), lipid profile, and renal function at baseline and at least weekly thereafter, together with blood pressure monitoring and periodic electrocardiography given the known cardiovascular risk of ponatinib.

On day 12, the patient developed abdominal pain, with a serum amylase concentration of 1376 U/L and a lipase concentration of 6777 U/L. Pancreatic computed tomography and abdominal ultrasonography showed no necrosis or fluid collection. Acute pancreatitis was graded as Common Terminology Criteria for Adverse Events (CTCAE) version 5.0 grade 3. Both agents were withheld, and the symptoms and biochemical abnormalities resolved with supportive care.

On day 28, complete blood count showed hemoglobin 60 g/L, platelets 29 × 10^9^/L, and white blood cells 1.76 × 10^9^/L. Bone marrow examination showed 0.5% blasts by morphology and 0.4% by flow cytometry, indicating a marked reduction in marrow blast burden. However, splenomegaly persisted, and *BCR*::*ABL1*/*ABL1* (IS) remained at 79%, indicating that the response was incomplete and not accompanied by molecular remission. Brain magnetic resonance imaging showed no radiological evidence of central nervous system involvement at that time; however, cerebrospinal fluid analysis was not performed, and normal imaging does not exclude occult leptomeningeal or CSF-based leukemic involvement. Treatment was resumed at reduced intensity with ponatinib 45 mg every other day plus asciminib 160 mg twice daily.

While preparation for haploidentical allogeneic hematopoietic stem cell transplantation was underway, additional interventions were undertaken because of massive splenomegaly and a persistent pulmonary lesion suspicious for mucormycosis despite approximately 3 months of amphotericin B therapy. The patient underwent pulmonary lobectomy on day 59, followed by splenic radiotherapy beginning on day 69. His son was identified as a haploidentical donor, and no donor-specific anti-HLA antibodies were detected.

**Figure 1:**
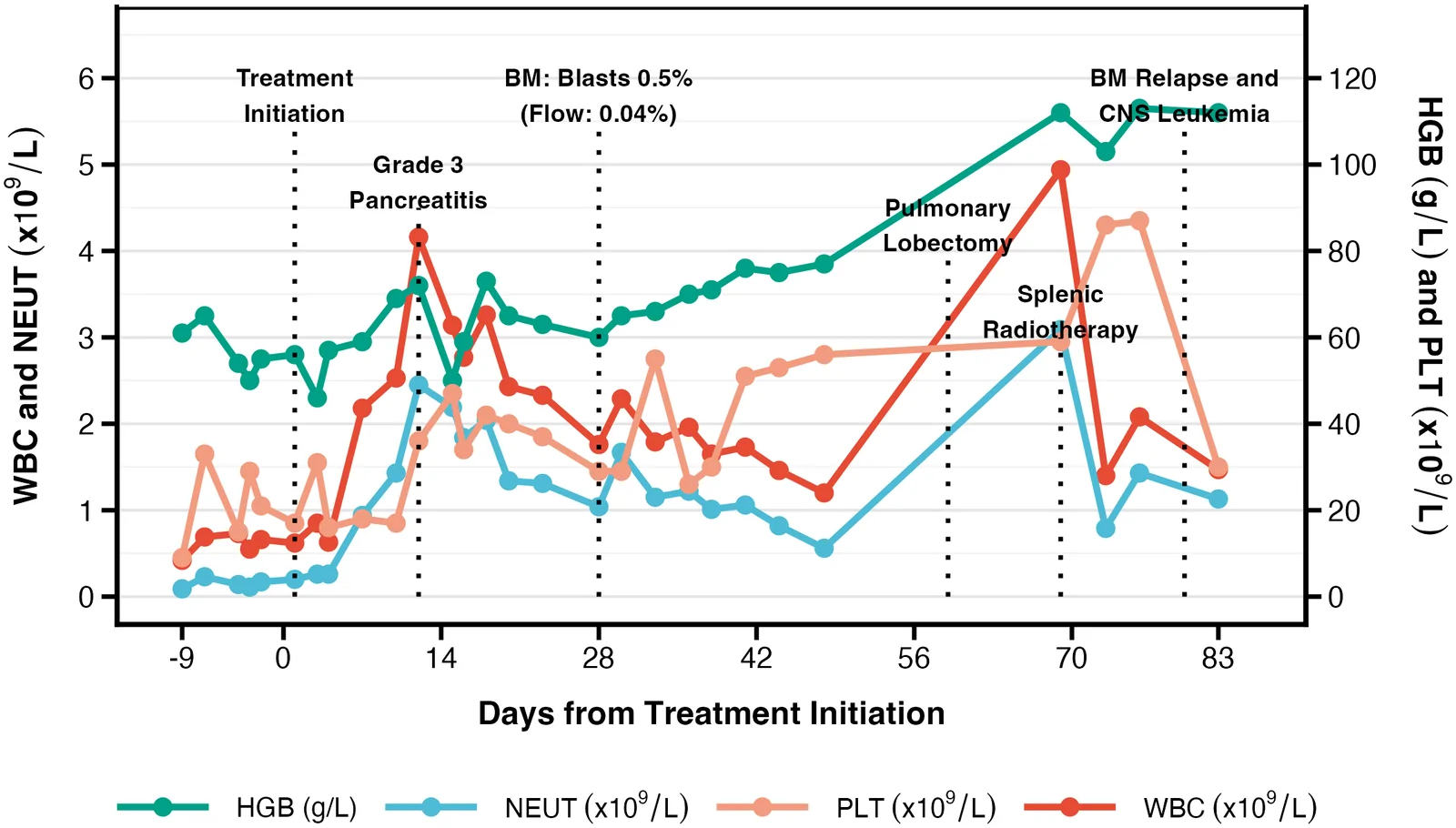
Changes in complete blood count parameters. Temporal changes in hematologic parameters during ponatinib–asciminib therapy. Key events include treatment initiation (Day 1), acute pancreatitis with treatment interruption (Day 12), marked reduction in marrow blasts (Day 28), pulmonary lobectomy (Day 59), splenic radiotherapy (Day 69), marrow relapse and CSF-confirmed CNS leukemia (Day 80). WBC and neutrophils are shown on the left y-axis; hemoglobin and platelets on the right y-axis.

On day 80, the patient developed headache. Contrast-enhanced brain magnetic resonance imaging was unremarkable; however, lumbar puncture showed a cerebrospinal fluid white blood cell count of 243 × 10^6^/L and protein of 533.5 mg/L. Cerebrospinal fluid flow cytometry demonstrated 73.68% myeloid blasts, confirming central nervous system leukemia. Concurrent bone marrow reassessment showed 13% blasts by morphology and 10.15% by flow cytometry, consistent with early relapse after the brief marrow response. The patient received three intrathecal treatments consisting of dexamethasone 5 mg, methotrexate 10 mg, and cytarabine 50 mg per procedure. He subsequently self-discharged, declined further therapy, and died of hemorrhage on day 115(**Figure 1** and **Table 1** summarize hematologic trends and the clinical timeline).

**Table 1:** Timeline of key clinical events, treatments, and *BCR*::*ABL1* mutations.

Date	Event	Treatment	*BCR*::*ABL1* mutations	Marrow blasts (%)	Other findings
2013	CML-CP	Imatinib 400 mg QD	—	<5	—
2013–2018	MMR maintained	Imatinib	—	—	*BCR*::*ABL1* IS ≤0.1%
Feb 2018	Loss of MMR	Imatinib	—	—	*BCR*::*ABL1* IS 1.98%
Jun 2020	Switch to 2nd-line TKI	Nilotinib 300 mg BID	—	—	—
Dec 2023	Myeloid blast phase	Nilotinib	F317L, H396R	9	Myeloid sarcoma
Feb–Mar 2025	Disease progression	Nilotinib	T315I, Y253H	17	—
Mar 2025	Switch to 3rd-gen TKI	Olverembatinib 40 mg QOD	—	—	Grade 4 myelosuppression
Jul 2025	Progressive disease	Olverembatinib	—	60	PB blasts 64%
Aug 2025	Intensive treatment	AZA+VEN+olverembatinib	—	17	Fungal infection, *K. pneumoniae* (BSI)
Sep–Oct 2025	Refractory disease	AZA+VEN+olverembatinib	T315I, Y253H, ASXL1	14→25	Complex karyotype, *BCR*::*ABL1* IS 207%
29 Oct 2025 (Day 1)	Combination therapy initiated	Ponatinib 45 mg QD + asciminib 200 mg BID	—	—	—
Day 12	Acute pancreatitis	Both agents held	—	—	Amylase 1376 U/L, lipase 6777 U/L
Day 28	Combination resumed; marked reduction in marrow blasts	Ponatinib 45 mg QOD asciminib 160 mg BID	—	0.5	0.4% by flow, *BCR*::*ABL1* IS 79%
Day 59	Pulmonary fungal infection	Both agents continued	—	—	Right pulmonary lobectomy
Day 69	Splenomegaly	Both agents continued	—	—	Splenic radiotherapy initiated
Day 80	BM relapse and CNS-leukemia	Both agents held	—	13	CSF blasts 73.68%, *BCR*::*ABL1* IS 89%

AZA, azacitidine; *BCR*::*ABL1* IS, *BCR*::*ABL1* international scale; BID, twice daily; BSI, bloodstream infection; CML-CP, chronic myeloid leukemia–chronic phase; CNS, central nervous system; CSF, cerebrospinal fluid; MMR, major molecular response; PB, peripheral blood; QD, once daily; QOD, every other day; TKI, tyrosine kinase inhibitor; VEN, venetoclax.

## 3 Discussion

The present case should be interpreted cautiously. Although a transient reduction in marrow blast burden was observed after the initiation of ponatinib plus asciminib, treatment exposure was brief and interrupted by pancreatitis, no molecular response was documented, and relapse occurred rapidly before allogeneic transplantation. Accordingly, this report does not establish efficacy and is best viewed as a hypothesis-generating clinical observation in a heavily pretreated patient with myeloid blast-phase CML.

At blast transformation, next-generation sequencing identified *BCR*::*ABL1* kinase domain mutations Y253H, T315I, and a low-level Y253_E255delinsHGK variant. Importantly, phasing analysis showed that Y253H and T315I were present on the same *BCR*::*ABL1* allele, confirming a true compound mutation rather than polyclonal co-occurrence. This distinction is clinically relevant because T315I-inclusive compound mutations, particularly those combining T315I with P-loop substitutions, have been associated with marked resistance to currently available TKIs in preclinical models (5, 10). The additional low-level Y253_E255delinsHGK variant suggests further clonal complexity, although its functional contribution cannot be determined from a single case. More broadly, this case supports the value of mutation profiling with phasing analysis — whether by long-read sequencing or other methods — in resistant or advanced-phase disease, where it may help distinguish therapeutically consequential compound mutants from separate subclones. Here, mutation detection relied on a targeted panel—efficient for routine use but restricted to predefined loci. Broader approaches (whole-exome, whole-genome, or whole-transcriptome sequencing) may reveal novel biomarkers and fusions beyond canonical loci, whereas long-read sequencing, as applied here, additionally resolves allelic phase and structural context. As these strategies become more accessible, their integration into the workup of advanced-phase or resistant disease may improve prognostic stratification and target identification.

Compound *BCR*::*ABL1* mutations are being recognized with increasing frequency as NGS-based approaches become more widely used, particularly in patients with resistance after second-line TKI therapy (11, 12). Among these, T315I-containing compound mutants are of particular concern. Byrgazov et al. showed that T315I combined with common P-loop substitutions, including Y253H, conferred substantial resistance to ponatinib in Ba/F3 models (10). Clinical evidence remains limited, although Jaeger et al. described the successful use of ponatinib combined with palbociclib in a patient with a *BCR::ABL1* compound mutation (13). Our case contributes an additional clinical observation to this limited literature by documenting a phased Y253H/T315I compound mutation in myeloid blast-phase CML.

The rationale for combining ponatinib with asciminib in this setting is mechanistically plausible. Ponatinib targets the ATP-binding site and retains activity against many single *BCR*::*ABL1* mutations, including isolated T315I, whereas asciminib binds the myristoyl pocket and inhibits *BCR::ABL1* through an allosteric mechanism (6–8).

On the basis of its approved indications, asciminib has been approved by the FDA and is recommended in NCCN guidelines for patients with T315I-positive CML at a dose of 200 mg twice daily, which informed the asciminib dose selected in our patient. As a single agent, however, asciminib retains activity against many but not all resistant variants, and its efficacy against T315I-inclusive compound mutations is limited when used alone, such that combination with an ATP-competitive inhibitor is required to restore effective suppression (9). This limitation provides the mechanistic rationale for combining asciminib with an ATP-competitive inhibitor such as ponatinib in compound-mutant disease. Dual targeting of these distinct sites has therefore been proposed as a strategy to suppress resistant compound mutants that are inadequately inhibited by either agent alone (9). Preclinical studies have demonstrated the additive or synergistic activity of ponatinib-allosteric inhibitor combinations, including ponatinib plus asciminib, against T315I-inclusive compound mutants and have suggested that effective suppression may be achievable at low-to-intermediate drug concentrations (9, 14–16). Recent clinical reports support this dual-targeting approach: Eide et al. showed that combined ponatinib and asciminib overcame BCR::ABL1 compound-mutant resistance (17), and Cheng and Li reported asciminib plus olverembatinib in a patient with blast-phase CML (18). Our case adds to this limited experience but, given the brief, interrupted exposure and rapid relapse, highlights that the efficacy, dosing, and tolerability of such combinations in blast-phase CML remain undefined.

In our patient, ponatinib 45 mg once daily plus asciminib 200 mg twice daily was selected empirically because of the aggressive biology of myeloid blast-phase CML, the presence of a phased *BCR*::*ABL1* Y253H/T315I compound mutation, and the urgent need for rapid cytoreduction before planned allogeneic transplantation. Ponatinib was started at 45 mg once daily to maximize early disease control in the setting of high marrow blast burden and aggressive disease tempo. Asciminib 200 mg twice daily was selected to attempt intensive dual-site *BCR*::*ABL1* inhibition in the context of a T315I-containing compound mutant, although the optimal dose of asciminib in combination with ponatinib in blast-phase CML is unknown. While preclinical data suggest that some T315I-inclusive compound mutants may be suppressible at lower drug exposures, these findings do not define an evidence-based starting dose in this clinical setting (9, 15). The subsequent development of grade 3 pancreatitis—a recognized complication of ponatinib therapy (19), although a contribution from asciminib cannot be excluded—required treatment interruption and dose reduction, underscoring the narrow therapeutic window of this dual-inhibition strategy. However, in rapidly progressive blast-phase disease with a high marrow blast burden, the need for prompt cytoreduction must be weighed against this potential benefit, and the optimal starting dose remains undefined in the absence of pharmacokinetic–pharmacodynamic data. A transient reduction in marrow blast burden was observed within 4 weeks, but the response remained incomplete, no molecular remission was documented, and relapse occurred rapidly before transplantation. Interpretation is further limited by the brief and interrupted treatment exposure, delayed earlier treatment optimization related to financial barriers, prior intervening therapies, advanced-phase high-risk biology, and the broader bridge-to-transplant context. Accordingly, the observed clinical improvement cannot be confidently attributed to ponatinib plus asciminib alone.

From a clinical perspective, this case is therefore best interpreted not as proof of efficacy but as a limited, hypothesis-generating observation suggesting possible short-term activity of dual ATP-site and allosteric inhibition in a highly refractory setting. A secondary practical lesson is that the loss of a molecular response milestone should prompt timely reassessment, including mutation testing and therapeutic review (3), because delays in treatment optimization may permit ongoing clonal selection and further narrow subsequent therapeutic options. In the present case, financial barriers delayed treatment switching by approximately two years after the loss of MMR, a period during which continued suboptimal *BCR*::*ABL1* suppression likely facilitated the stepwise acquisition of resistance mutations and clonal evolution toward blast transformation. This observation underscores the broader importance of equitable and timely access to second-line TKIs, as delays in treatment optimization may permit progressive mutational complexity that ultimately limits salvage options. For patients with advanced-phase CML and compound *BCR*::*ABL1* mutations, allogeneic transplantation remains the only potentially curative strategy (3), and targeted combinations should presently be considered individualized bridge approaches rather than established standard therapy. Future progress will likely depend on collaborative case series or registries, serial molecular monitoring to define clonal dynamics during treatment, and pharmacokinetic–pharmacodynamic studies to identify dosing strategies that better balance activity with tolerability.

## 4 Conclusion

We report a transient reduction in marrow blast burden temporally associated with ponatinib plus asciminib in myeloid blast-phase CML harboring a phased Y253H/T315I *BCR*::*ABL1* compound mutation. Given the brief and interrupted exposure, absence of molecular remission, and rapid relapse, this case should not be interpreted as evidence of efficacy. Rather, it provides a cautious, hypothesis-generating clinical observation that may inform future study of individualized dual ATP-site and allosteric inhibition as bridge therapy in highly resistant compound-mutant CML.

## Data Availability

The original contributions presented in the study are included in the article/**Supplementary Material**. Further inquiries can be directed to the corresponding authors.
